# Motion-Status-Driven Piglet Tracking Method for Monitoring Piglet Movement Patterns Under Sow Posture Changes

**DOI:** 10.3390/vetsci12070616

**Published:** 2025-06-24

**Authors:** Aqing Yang, Shimei Li, Shuqin Tu, Na Han, Lei Zhang, Yizhi Luo, Yueju Xue

**Affiliations:** 1 College of Computer Science, Guangdong Polytechnic Normal University, Guangzhou 510665, China; yangaqing@gpnu.edu.cn (A.Y.); hannagdut@126.com (N.H.); lei_power@hrbeu.edu.cn (L.Z.); 2 AI Middle Platform Department, Guangzhou Kingmed Diagnostics Group Co., Ltd., Guangzhou 510005, China; 13600466344@163.com; 3 College of Mathematics and Informatics, College of Software Engineering, South China Agricultural University, Guangzhou 510642, China; tushuqin@163.com; 4 Institute of Facility Agriculture, Guangdong Academy of Agricultural Sciences, Guangzhou 510640, China; 5 College of Electronic Engineering, South China Agricultural University, Guangzhou 510642, China

**Keywords:** social relationships, behavioral patterns, stress behavior recognition, multi-object tracking, hierarchical matching mechanism

## Abstract

Understanding how piglets move around sows during posture changes is crucial for their safety and healthy growth. Automated monitoring can reduce farm labor and help prevent accidents like piglet crushing. Current methods (called Joint Detection-and-Tracking-based, abbreviated as JDT-based) struggle with problems like misidentifying piglets or losing track of them due to crowding, occlusion, and shape changes. To solve this, we developed MSHMTracker, a smarter tracking system that introduces a motion-status hierarchical architecture to significantly improve tracking performance by adapting to piglets’ motion statuses. In MSHMTracker, a score- and time-driven hierarchical matching mechanism (STHM) was used to establish the spatio-temporal association by the motion status, helping maintain accurate tracking even in challenging conditions. Finally, piglet group aggregation or dispersion behaviors in response to sow posture changes were identified based on the tracked trajectory information. Tested on 100 videos (30,000+ images), our method achieved 93.8% tracking accuracy (MOTA) and 92.9% identity consistency (IDF1). It outperformed six popular tracking systems (e.g., DeepSort, FairMot). The mean accuracy of behavior recognition was 87.5%. In addition, the correlations (0.6 and 0.82) between piglet stress responses and sow posture changes were explored. This research showed that piglet movements are closely related to sow behavior, offering insights into sow–piglet relationships. This work has the potential to reduce farmers’ labor and improve the productivity of animal husbandry.

## Simple Summary

This study first proposed an enhanced JDT-based motion-status-driven hierarchical piglet tracking method, named MSHMTracker, to address the challenges of identity switches and target losses during piglet tracking caused by crowding, occlusion, and shape deformation. In MSHMTracker, a score- and time-driven hierarchical matching mechanism (STHM) was proposed to establish the spatio-temporal association by motion status. Then the piglets’ motion information obtained from MSHMTracker was analyzed to recognize piglet groups’ stress (aggregation/dispersion) behavior responses to the sow’s posture changes. Later, the correlation between the movement patterns (aggregation and dispersion) of piglets and the posture transitions (upwards and downwards) of the sow was explored. MSHMTracker achieved improvements of 2.1% and 2.3% in IDF1 and MOTA, respectively, compared to the baseline model. The average accuracy of piglets’ stress (aggregation/dispersion) behavior recognition reached 87.49%. And the correlation values between piglets’ aggression and dispersion and the sow’s upward and downward posture changes were 0.6 and 0.82, laying the foundation for research on the social relationships and behavioral patterns between lactating sows and piglets.

## 1. Introduction

Currently, a large number of researchers are dedicated to selective breeding programs for improving pig productivity, disease resistance, and physical and mental health through observing and exploring the social relationships and behavioral patterns between lactating sows and piglets [1,2] since the traits of pigs can be reflected directly by their individual, group, and social behaviors. For example, piglet groups exhibit aggregation or dispersion behavior in response to sow posture transitions [3,4,5]. When the sow lies down to nurse, piglets will gather around her udders. When the sow stands up or moves, piglets will disperse to avoid harm. These behaviors help reduce piglet crushing and ensure the safety and healthy growth of piglets [6,7]. However, the acquisition of this information mainly relies on manual observation, which is time-consuming, labor-intensive, and prone to errors. Therefore, the automatic monitoring of sow postural changes and piglet movement patterns may help reduce piglet crushing, which is necessary and crucial for selective breeding programs to further promote the development of the pig farming industry.

In automatic monitoring systems, non-contact computer-vision-based monitoring is more economical, convenient, and beneficial for animal welfare protection compared to wearable- or embedded-sensor-based monitoring. This non-contact and efficient computer vision technology has been widely used for monitoring pig behaviors such as feeding [8,9], moving [10,11], and abnormal [12,13,14] and other behaviors [15,16]. However, there has been limited research on automatic recognition and on exploring the social relationships and behavioral patterns between the mother sow and her piglets. And these studies mainly focused on the nursing behavior [17,18] and nosing behavior [19] of pigs, which are very different from our study on stress behavior in pig groups in response to a sow’s postural changes. These approaches focused on spatial–temporal localization through segmentation and keypoint detection and were not concerned with motion trajectories. For example, nursing was defined by the piglet location in the nursing zone, whereas nosing behavior was assessed through piglet–sow distance measurements. We observed that piglets’ stress responses to sow postural changes involve more complex kinematic signatures: movement patterns relative to the mother sow. For instance, when piglets are playing around the sow, their spatial positions may change, but the piglets do not show a significant direction (toward/away from the mother sow). However, this study still faced challenges in behavioral inconsistency within the group. Also, the interaction between individual sow and piglet responses in the movement direction needs to be explored. Therefore, a new method that can capture the location, movement direction, and piglet ID was the focus of this work.

Multi-object tracking (MOT) technology conducts target recognition and tracking in video scenes, capturing the target category, position, motion direction, and trajectories, which are critical for accurate behavior recognition, facilitating livestock farmers’ decision-making [20]. Recently, MOT has achieved state-of-the-art performance in behavior recognition in pigs [16,21,22]. Most studies use a tracking-by-detection (TBD) method, where detection and tracking are conducted in sequence by two independent modules. So, tracking highly depends on the accuracy of detections and requires a complex data association algorithm to link detections over time. Currently, YOLO-based networks [16,23] constitute the mainstream detection method due to YOLO’s excellent detection performance, and they are commonly combined with Byte [24] or Sort-like trackers [25] for multi-target tracking. For example, YOLOv5 was used to detect individual pig behaviors, and then the Byte method was used to track the behaviors of individual pigs [26]. The detection network was enhanced by integrating an efficient attention mechanism into the backbone network, followed by the Byte algorithm to track the movement trajectories of individual pigs [15]. YOLO v7 and DeepSORT were used to detect and track pigs in videos to model the pig behavior patterns for health monitoring [27]. YOLOv4 and YOLOv7 were used to locate ear-biting regions and associated these detections with DeepSORT and centroid tracking algorithms [28].

Compared with TBD paradigms, the joint detection and tracking (JDT) paradigm integrates detection and tracking into a single network, potentially reducing the dependence of the tracker on the detector. The key in JDT is learning features from multi-frame data, simultaneously capturing spatial and temporal features, which may dig out potential association clues about the targets between adjacent frames. For instance, Krhenbühl et al. [29] introduced CenterTrack, a point-based framework designed to simplify object association across time. Zhang et al. [30] introduced FairMOT, a point-based framework similar to CenterTrack, enhancing object association through Re-ID feature recognition. Although these methods demonstrate simplicity and effectiveness, they still have limitations in associating lost targets with the previous trajectory and struggle to locate targets in complex scenes, such as those with occlusion and crowding. In addition, the behavioral analysis of sows and piglets within free-range pens is challenged by certain characteristics: (1) occluded, densely crowded, and clustered piglets; (2) similar appearance and shape deformation; (3) irregular movement and interaction among pigs. These factors can exacerbate target loss or ID switching.

To address these challenges and better explore the social relationships and behavioral patterns between lactating sows and piglets, we proposed a JDT-based MOT method, named MSHMTracker, to analyze stress behaviors in piglet groups in response to a sow’s posture changes. In the matching process, we introduced a score-driven and time-driven hierarchical matching mechanism (STHM) to extract subtle yet useful clues for associations between low-score, occluded, and reappearing objects and previous tracklets, ensuring trajectory completeness. Finally, by integrating the target trajectory and movement direction, we explore the social relationships and behavioral patterns between lactating sows and piglets.

## 2. Materials and Methods

### 2.1. Data Collection

The data on lactating sows and piglets were collected from a Lejiazhuang farm in Foshan city, Guangdong province, China. The sows belong to a local breed officially named “Small-ear Spotted pig”, which have small spots on their bodies. The piglets are completely black and are hybrid offspring from crosses between Small-ear Spotted pigs and either Tibetan Xiang pigs or Duroc pigs. In order to develop a robust tracking algorithm, the experimental data were captured in different time batches, under different lighting conditions, and using different cameras. The experimental data were captured from 11 pens, resulting in a total of 11 sows and over 100 piglets, where piglets in different pens were different ages.

From the recordings of each pen, we selected 10 30-s videos, resulting in 110 videos/33,000 images in total. Those videos with shape deformation, occlusion, crowding, or variable lighting conditions were also selected. These videos were divided into the training set, validation set, and test set. Dataset partitioning is described in Table 1, which shows that the training set contained 72 videos/21,600 images from 8 pens, the validation set contained 8 videos/2400 images, and the testing set contained 30 videos/9000 images from the other 3 pens.

For subsequent supervised learning and performance validation, the ground truth (GT), including the ID and position information of each pig in a video, was labeled by using the DarkLabel v1.3 software. It should be noted that the consistency of the corresponding pig ID in all frames of each video must be carefully maintained.

**Figure 1:**
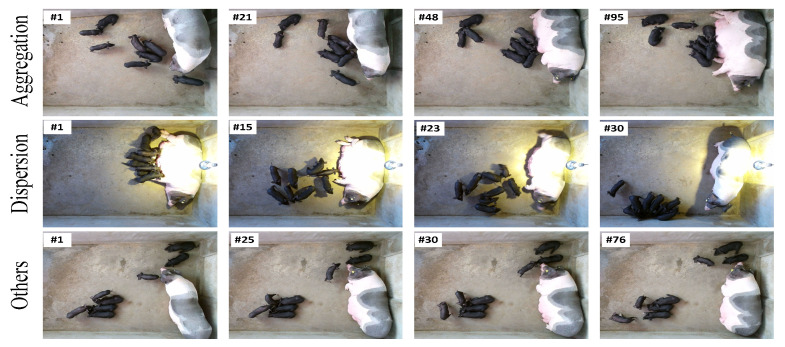
Examples of aggregation, dispersion, and other behaviors of piglets in response to the sow’s upward, downward, and rolling posture changes.

To explore the social relationships and behavioral patterns between lactating sows and piglets, we manually extracted 100 videos of sow posture changes to analyze piglet groups’ stress responses to sows’ posture changes. The dataset comprised 100 annotated sow-posture-change episodes (52 upward/34 downward/14 other), with the corresponding piglet group behaviors quantified as 41 aggregation events, 36 dispersion events, and 23 undefined responses, as described in Table 1. Figure 1 illustrates the aggregation, dispersion, and other behaviors of piglets in response to sows’ upward, downward, and rolling posture changes. The number prefixed with “#” in the upper left corner of each image represents the frame ID.

### 2.2. The Overall Framework of Behavior Exploration

**Figure 2:**
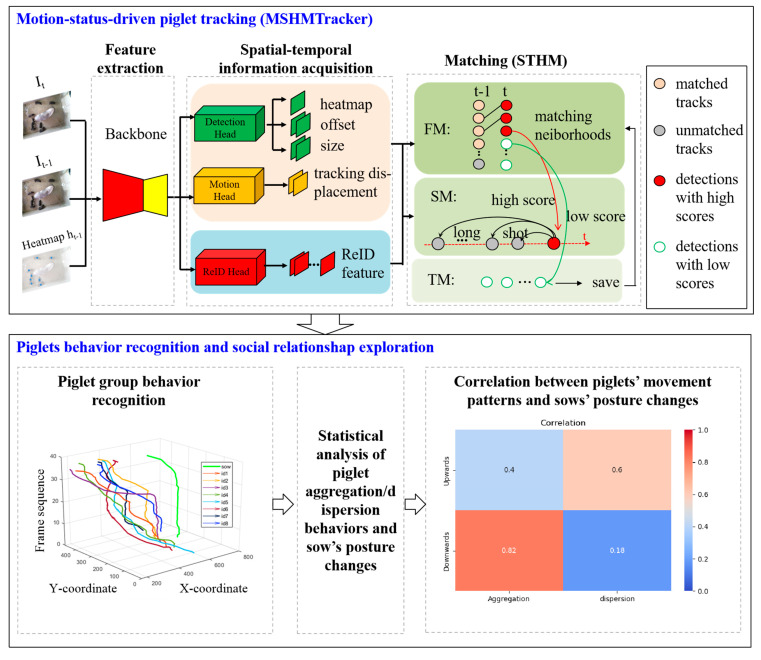
Overview of behavior exploring framework.

Figure 2 presents the overall framework of the proposed methodology for exploring the social relationships and behavioral patterns between lactating sows and piglets based on a tracking method. It consists of two main components: motion-status-driven piglet tracking and tracker-based social behavior exploration.

### 2.3. Motion-Status-Driven Piglet Tracking

#### 2.3.1. Problem Formulation

JDT aims to associate identical objects across video frames to obtain complete motion trajectories using a single network. It establishes spatio-temporal relationships between multi-frame images by learning from consecutive frame data, which can be formulated as

(1) Dt,M=FJDT([It,It−1]),Tt=Gmat(Dt,M),

where F_JDT_(∙) represents the JDT tracker. $I_{t\;}and{\;I}_{t-1}$ represent the frames at time t and time t − 1, respectively. $D_{t}$ represents the detection results of frame $I_{t}$, which usually contains the position and size of the bounding box. M represents the inter-frame motion vector of objects in frame $I_{t}$. The JDT method typically employs a simple matching method G_mat_(∙) such as a greedy or Hungarian algorithm to perform data association.

JDT can capture potential temporal associations, reducing the reliance on detection quality and enhancing its robustness in complex environments. However, in a real pig farm environment, JDT still struggles with ID switching and mismatching due to shape deformations, crowding, overlapping, and occlusion. Therefore, this paper proposes a motion-state-driven hierarchical matching model for piglet tracking (MSHMTracker), building upon the foundation of JDT. MSHMTracker takes a step further by incorporating the ReID model and introducing a systematic and hierarchical matching strategy based on the decomposition of the object motion process. The MSHMTracker can be formulated as follows:

(2) Dt,M,At=FJDT([It,It−1],ht−1),Tt=Gstm(Gmat(Dt,M),At),

where $h_{t-1}$ is the heatmap produced from F_JDT_(∙). $A_{t}$ represents the appearance feature of objects, generated by the proposed ReID head. During matching, a score–time-weighted appearance similarity matching method $G_{stm}$ is introduced to enhance the tracking performance for reappearing objects.

#### 2.3.2. Overall Architecture of MSHMTracker for Piglet Tracking

As shown in Figure 1, MSHMTracker consists of three key modules: cross-temporal feature extraction, spatio-temporal information acquisition, and score–time-driven hierarchical matching mechanism (STHM). MSHMTracker follows the JDT paradigm and is built upon the anchor-free CenterNet, using Deep Layer Aggregation (DLA) as its backbone network. MSHMTracker takes the current input I_t_, the previous frame I_t−1_, and the heatmap h_t−1_ as inputs, and it outputs an object response heatmap, object center offsets, object size, object tracking displacement, and the object ReID appearance feature. This information is fed to the STHM matching module for target association, and the target trajectory is established by the proposed hierarchical data association mechanism.

#### 2.3.3. Cross-Temporal Feature Extraction

MSHMTracker takes the current frame $I_{t}\in R^{W\times H\times C}$, the previous frame $I_{t-1}\in R^{W\times H\times C}$, and a heatmap h_t−1_ rendered from the prior tracks’ center points {P_0_, P_1_, ∙∙∙, P_n_} as inputs. Here, the pair of frames $I_{t}$ and $I_{t-1}$ enables the network to estimate the changes in the scene and potentially recover the occluded objects at time t that were visible at time t − 1. The heatmap h_t−1_ of prior tracklets helps the network learn to repeat the predictions from the prior frame and simplifies MSHMTracker to match objects across time. As shown in Figure 1, we applied the encoder–decoder network of DLA-34 with deformable convolution layers to the backbone of CReIDTrack for feature extraction.

#### 2.3.4. Spatio-Temporal Information Acquisition

After feature extraction, these features were fed into a spatio-temporal information acquisition module to obtain object spatial position, appearance, and temporal motion information. Unlike the mainstream JDT-based algorithms, a ReID branch was introduced to achieve trajectory recovery and alleviate the target loss and ID switch. Also, the ReID results will be transferred to the score–time-driven hierarchical matching stage, which will be described in Section 2.3.5. In general, the spatio-temporal information acquisition process consists of three key branches: the detection head, motion head, and ReID head. Each branch is realized by a task head that is composed of a 3 × 3 convolution layer, a Relu layer, and a 1 × 1 convolution in sequence.

The ReID branch is used to obtain the appearance features of piglet targets, which can distinguish piglets and will be used to re-identify the target ID during the matching stage to match the same object over time. As with the other branches, the ReID branch consists of two convolutional layers and a Relu layer, with an output feature size of $Dim\times \frac{W}{4}\times \frac{H}{4}$, where W and H are the length and width of the input image, respectively, Dim = 64 is the number of output feature channels. Here, we extract an identity feature vector at the object $d_{i}$ center on the heatmap and map it to a class distribution vector ${\hat{Q}}^{i}(m)=\{q(m),m\in (1,M)\}$, where M is the number of categories, and q(m) is 0 or 1, indicating the id flag of category m. This mode aims to obtain identity features without increasing the computational overhead. The one-hot representation of the ground-truth label is denoted as $Q^{i}(m)$. The loss function of ReID $L_{reid}$ is defined as the cross-entropy loss:

(3) $$L_{reid}=\frac{1}{N}\sum_{i=1}^{N}\sum_{m=1}^{M}Q^{i}(m)log(q(m))$$

#### 2.3.5. Score–Time-Driven Hierarchical Matching (STHM)

We observe that the JDT paradigm usually employs a simple greedy matching algorithm based on the displacement of the target center across frames and ignores ID recovery during matching. Under the condition of target deformations, crowding, overlap, and occlusion, the typical matching method of JDT can lead to target loss and ID switching during piglet tracking. Existing ReID methods employ appearance information to help restore missing objects, but this does not work on occluded and deformable targets. To improve the matching ability, especially for occluded and reappearing objects, an STHM module was proposed that utilizes hierarchical matching based on the motion status of the object.

**Figure 3:**
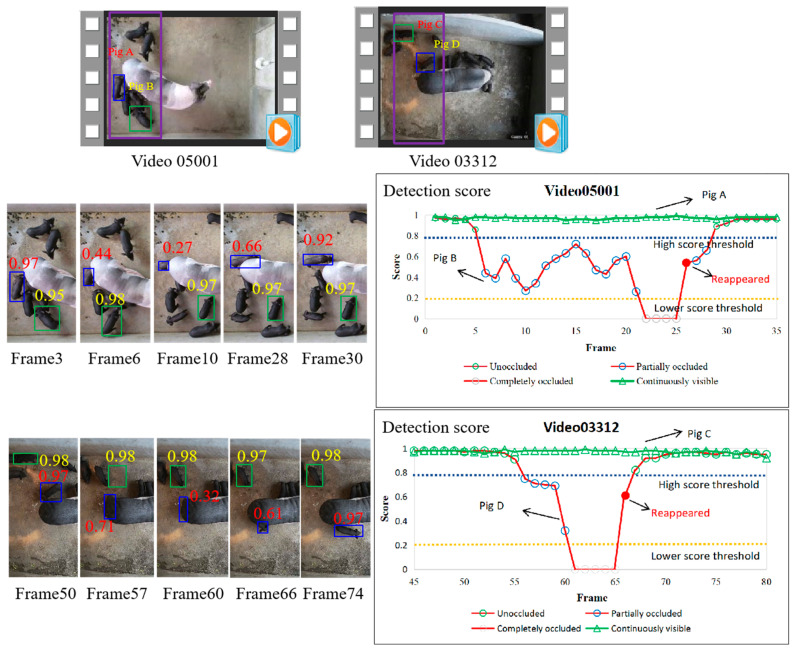
Variation in detection score of occluded objects over time.

To systematically analyze the motion status of piglets, we performed a thorough observation and analysis of our experimental scenario and the locomotion patterns of piglets. We randomly selected two scenes and recorded the detection scores of occluded and unobstructed objects over time. As shown in Figure 3, blue bounding boxes represent continuously visible piglets, and green ones denote piglets that become gradually occluded and then reappear. It is not difficult to observe that when the object is partially occluded, the detection score decreases significantly. When the object reappears, the detection score increases noticeably. Usually, during the process of target tracking, occluded objects frequently suffer from missed detection due to reduced confidence scores, whereas reappearing objects tend to cause ID switches due to positional changes.

**Figure 4:**
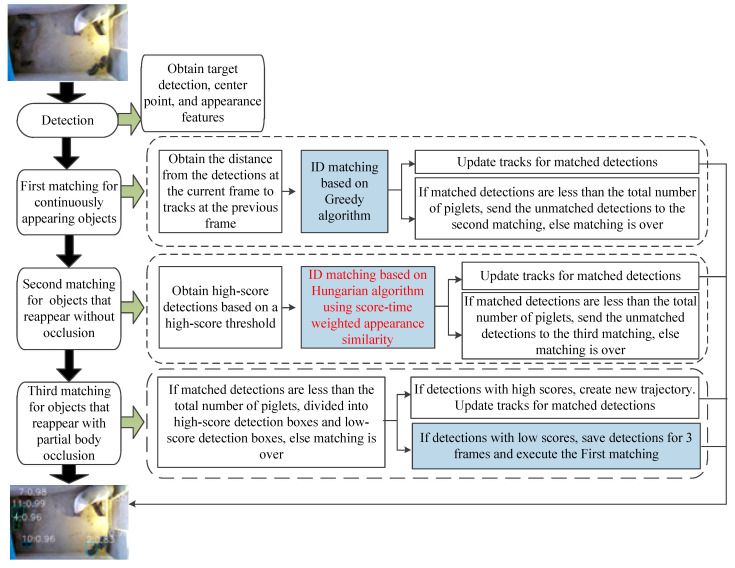
The matching process of STHM.

To address this problem, based on the pig movement status and detection score, we classified objects into three categories: continuously visible, partially occluded, and reappeared. Based on the characteristics of these three types of objects, a score–time-driven hierarchical matching mechanism (STHM) was proposed. It first performs matching for the first type of object by the inter-frame center distance, then associates the objects that reappear without occlusion by the score–time-weighted appearance similarity, and finally associates the objects that reappear with partial occlusion by a saving-and-iteration strategy. The matching process of STHM is shown in Figure 4.

(1) First matching for continuously appearing objects

When piglets maintain continuous visibility in video sequences (whether fully visible or partially occluded), reliable ID matching is achievable through the inter-frame center point distance computation of piglets within the point-based JDT framework. Compared with the IoU metric, the center point distance is not sensitive to the size variations caused by occlusion, clustering, and deformation. During the matching process, the unmatched detections tend to be newly appearing or reappearing targets, while the unmatched tracklets may be caused by target loss.

(2) Second matching for reappeared objects

**Figure 5:**
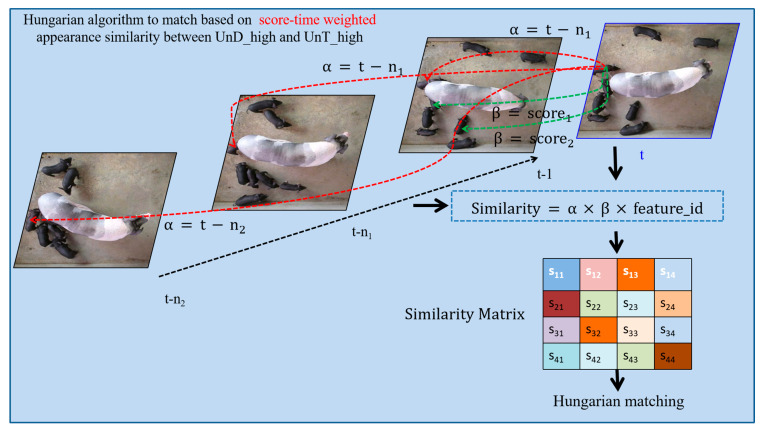
The matching process of score–time-weighted appearance similarity. The red dashed lines indicate target matching across different time sequences, while the green dashed lines represent target matching across different spatial locations. The black dashed line represents the time series. Black arrows indicate methodological workflow. In the similarity matrix, color-coded cells indicate varying degrees of similarity.

In this section, score–time-weighted appearance similarity is proposed for reappearing objects. The similarity computation method is illustrated in Figure 5. During tracking, any unmatched tracklets are retained in their original time sequence while preserving their confidence scores. Specifically, if the number of piglets currently matched is lower than the total count, the unmatched detections and unmatched tracklets are associated through the following steps:

Step 1: Calculating appearance similarity according to the following formula (see formula in blue dashed box, Figure 5):

(4) Appearance similarity=α×β×feature_id,

where $\alpha =t-n_{i}$ indicates the duration (in frames) that a trajectory has remained unmatched. β presents the confidence score. feature_id is the reID feature obtained from the ReID head.

Step 2: Performing ID matching by using a Hungarian matching algorithm based on appearance similarity.

(3) Third matching for newly emerging or reappearing piglets with significantly altered appearance

If the number of currently matched piglets is lower than the total count, the unmatched detections, unDets2, will be divided into unDets2_high and unDets2_low based on a high-score threshold ${Score}_{h}$. unDets2_high is likely to be newly emerging piglets, creating a new trajectory. unDets2_low tends to be reappearing objects with significantly altered appearance. These detections will be temporarily reserved for 3 frames and proceed to the first matching based on the distance metric. If no successful matches occur during this period, these detection boxes are deleted from the tracking pipeline.

### 2.4. Behavioral Monitoring

**Figure 6:**
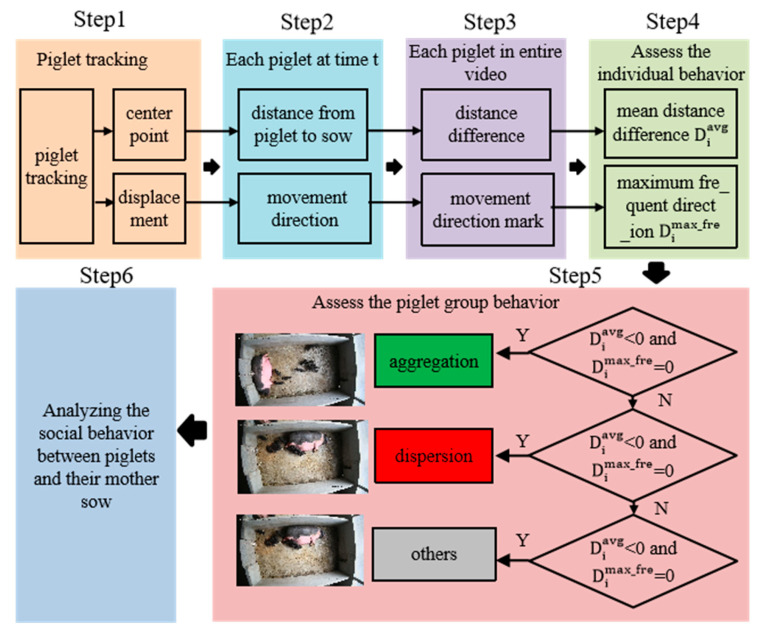
The process for recognizing the aggregation and dispersion behaviors of piglet groups.

For the process of dispersion/aggregation behavior recognition, a spatial range of stress occurrence was defined, and it was specified as a circular domain centered at the sow’s centroid, with a radius equal to the sow’s body length. Then the recognition and statistical analysis of piglet aggregation/dispersion behaviors were conducted within the specified spatial range. The recognition process is shown in Figure 6.

Step 1: Acquire the i-th piglet’s centroid coordinates $\left(x_{i}^{t},y_{i}^{t}\right)$ and motion displacement $\left({dx}_{i}^{t},{dy}_{i}^{t}\right)$ at time t from MSHMTracker.

Step 2: Calculate the Euclidean distance ${CD}_{i}$ between the sow’s centroid $\left(x_{s}^{t},y_{s}^{t}\right)$ and the piglet’s centroid $\left(x_{i}^{t},y_{i}^{t}\right)$ of the i-th piglet at time t. Through this process, obtain the center distance ${CD}_{i}=\{{CD}_{i}^{1},{CD}_{i}^{2},...,{CD}_{i}^{t}\}$ throughout the entire video clip. Denote the distance difference as $D_{i}^{t}={CD}_{i}^{t}-{CD}_{i}^{t-1}$: if $D_{i}^{t}\;$> 0, the piglet is approaching the sow; otherwise, it is moving away.

Step 3: Calculate the piglet’s motion direction ${Op}_{i}^{t}$ and the piglet-towards-sow direction ${Os}_{i}^{t}$ at time t.

**Figure 7:**
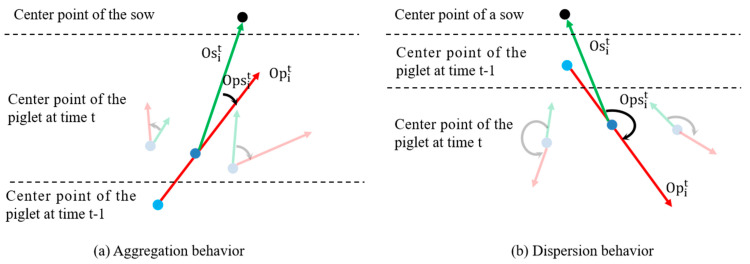
Analysis of the angle ${Ops}_{i}^{t}$ between a piglet’s movement direction ${Op}_{i}^{t}$ and the direction towards the sow’s ${Os}_{i}^{t}$ under aggregation (**a**) and dispersion (**b**) behaviors. The red arrows represent the piglet’s motion direction ${Op}_{i}^{t}$. The green arrows represent the piglet-towards-sow direction ${Os}_{i}^{t}$. The black arrows represent the angle between ${Os}_{i}^{t}$ and ${Op}_{i}^{t}$.

Denote the angle as ${Ops}_{i}^{t}=\left|{Op}_{i}^{t}-{Os}_{i}^{t}\right|$. When a piglet is moving towards its mother sow, ${Ops}_{i}^{t}$ is relatively small. Conversely, when a piglet is moving away from its mother sow, ${Ops}_{i}^{t}$ is bigger, as shown in Figure 7. We specify that if ${|Ops}_{i}^{t}|<$ 75 degrees, the piglet exhibits movement towards the sow at time t, marking it as ‘0’; if 105 degrees $<{Ops}_{i}^{t}<$ 255 degrees, the piglet exhibits movement away from the sow at time t, marking it as ‘1’; else it is marked as ‘2’. Based on this, obtain the motion direction mark $O_{i}^{t}$ of the i-th piglet.

Step 4: Calculate the mean distance difference $D_{i}^{avg}$ and the most frequent movement direction of piglets $O_{i}^{max\_fre}$ to assess the individual behavior of the i-th piglet in the video. These two metrics have good robustness to the movement noise from crowding and sudden movements. If $D_{i}^{avg}\;$< 0 and $O_{i}^{max\_fre}\;$ = 0, the piglet is gathering towards the sow, marking the piglet’s behavior $B_{i}$ as 0; if $D_{i}^{avg}\;$> 0 and $O_{i}^{max\_fre}\;$= 1, the piglet is moving away from the sow, marking the piglet’s behavior $B_{i}$ as 1; otherwise, it is marked as 2. At this point, we have obtained the individual behaviors of all the pigs and stored them in the set $B_{i}=\{B_{1},B_{2},...,B_{N}\}$, where N is the number of piglets.

Step 5: Conduct a statistical analysis of pig group behavior from $B_{i}$. If the value of $B_{i}\;$= 0 is more than $\frac{N}{2}$, the pig group behavior is considered aggregation; if the value of $B_{i}$ = 1 is more than $\frac{N}{2}$, the pig group behavior is considered dispersion; otherwise, it is considered other behavior.

Step 6: Explore the social behavior by analyzing the aggregation and dispersion behaviors of piglet groups under different states of sows’ posture changes and explore the relationships and behavioral patterns between sows and piglets.

### 2.5. Evaluation Metrics

MOTA (Multi-Object Tracking Accuracy), IDF1 (Identity F1 Score), ID Switch (IDs), FP (False Positive), and FN (False Negative) were selected to evaluate the performance of the piglet tracking model. The MOTA metric evaluates a tracker’s performance in object detection and trajectory maintenance. IDF1 emphasizes the tracker’s ability to maintain consistent target identities during re-identification scenarios. IDs counts the identity switches during tracking. In addition, the tracking rate was used to evaluate our method’s performance in each piglet tracking. The tracking rate represents the ratio of frames in which the target was successfully tracked to the total frames with its ground-truth presence. These metrics are widely recognized and commonly used in computer vision for multi-object tracking.

### 2.6. Experimental Details

Three experiments were conducted: piglet tracking, aggregation and dispersion behavior recognition, and exploration of the relationship and behavioral patterns between the sows’ posture changes and piglets’ group behaviors. During the training phase of the piglet tracking model, the model was trained using the original Adam optimizer. The batch size, learning rate, and epoch were set to 16, 0.0001, and 120, respectively. The default input resolution for our pig images is 1920 × 1080. The images were resized to 960 × 544. We set the high-score threshold ${Score}_{h}=0.6$ and the low score ${Score}_{l}=0.2$. All experiments were run using an Nvidia TITAN V GPU with the PyTorch 3.8 framework.

## 3. Results

### 3.1. The Results of Piglet Tracking

#### 3.1.1. Comparison of Different Improvement Strategies Proposed in Our Tracking Model

MSHMTracker integrates a ReID module within the JDT framework and uses a hierarchical data association strategy based on object motion status. Consequently, ablation experiments on the hierarchical strategies were conducted. The results are presented in Table 2. By using the FM (the first matching) and SM (the second matching), the method exhibits significant improvements in IDF1 (+1.0%) and MOTA (+2.5%), and we owe this improvement to the second matching’s ability to restore the target trajectory for the reappearing targets. The third matching (TM) exhibits a minor improvement of 0.4% in IDF1 and a reduction of 0.3% in MOTA. This is mainly because the TM specifically handles reappearing objects with partial occlusion that account for a small proportion of the dataset.

We also calculated the tracking rate for each piglet in all of the test videos. The results are presented in Table 3. It can be seen that our module exhibits a good performance (with a mean tracking rate of 0.98) for each piglet tracking. Only a small number of pigs have a low tracking rate (e.g., 0.58, 0.69). These pigs were usually located in very dark areas or had most of their bodies covered, which makes our model unable to recognize them.

#### 3.1.2. Comparison with Different Tracking Methods

We compared MSHMTracker with several mainstream methods, including Motdt, FairMot, DeepSort, ByterTrack, Sort, and Centertrack. The results are presented in Table 4. Note that all methods were trained on our training set, and the results were obtained from our test set. Our method demonstrates superior performance among all compared methods. Compared to the classic method Motdt, MSHMTracker achieves 37.8% (55.1% → 92.9%) improvement in IDF1 and 9.1% (84.7% → 93.8%) improvement in MOTA. Compared to the baseline method Centertrack, MSHMTracker achieves 2.1% (90.8% → 92.9%) improvement in IDF1 and 2.3% (91.5% → 93.8%) improvement in MOTA. However, MSHMTracker has no improvement in IDs compared to Centertrack; one of the possible reasons is that the ID-switched targets are mostly reappearing targets and undergo severe deformation, which poses a great challenge to the tracking algorithm.

### 3.2. Study on Behavioral Patterns Between Lactating Sow and Piglets

#### 3.2.1. The Results of Aggregation and Dispersion Behavior Recognition of Piglet Groups

To explore the social relationships and behavioral patterns between lactating sows and piglets, we first identified the stress responses of piglets to changes in sow posture by using the method described in Section 2.4. Here, the stress responses mainly include aggregation and dispersion behaviors. In addition, we have also categorized another type of behavior. The “other behaviors” category refers to instances where piglets showed little response to a sow’s posture transition. This typically occurred when piglets were either sleeping or spatially distant from the sow, indicating reduced sensitivity to the sow’s movements. In these situations, the piglets show almost no response to stress changes in the posture of the sow.

The experiments were conducted on a total of 100 episodes containing 42 aggregation events and 36 dispersion events. The recognition results are shown in Table 5. For the 41 episodes of aggregation, 37 were recognized correctly, resulting in an accuracy of 95.24%. For the 36 videos of dispersion, only 26 were recognized, and 9 were misclassified as other behaviors. The main reason is that piglets tend to briefly scatter and then re-cluster during sow posture transitions. Generally, they rapidly disperse to maintain a safe distance during the sow’s posture changes, followed by re-clustering to resume suckling proximity, maintain body temperature, or gain a sense of security. Under these conditions, the recognition algorithm tends to misclassify dispersion as other behaviors. This occurs because dispersion/aggregation behaviors are modeled based on the mean distance difference and the most frequent movement direction of piglets across the entire video. The later irregular motions may weaken the characteristic features of dispersion.

#### 3.2.2. The Exploration of Piglet Group Behavior Responses to Sow Posture Changes

The association between piglets’ aggregation/dispersion behaviors and sow posture changes is explored in this section. The mother sow’s posture changes were manually classified into downward posture changes, upward posture changes, and rolling posture changes. The statistical results for the video count and 95% confidence interval (CI) of piglet aggregation/dispersion behaviors during sow posture transitions are shown in Table 6.

**Figure 8:**
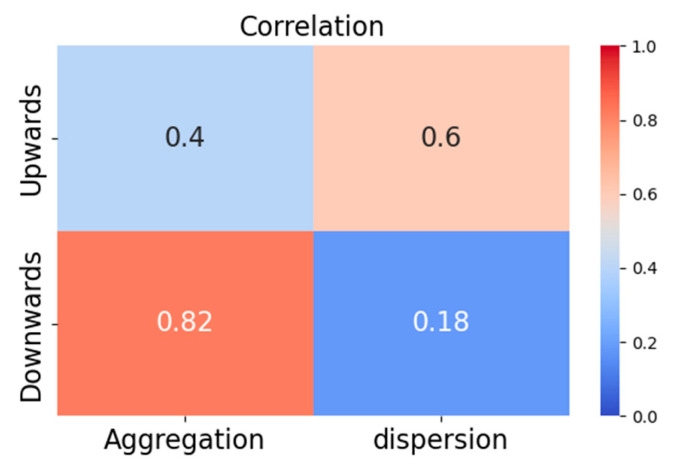
The correlation between piglets’ movement patterns and sows’ posture changes.

As shown in Table 6, piglet aggregation/dispersion behaviors varied significantly under different posture changes. Specifically, of 52 videos with upward posture changes in sows, dispersion behavior was observed in 27 cases, and aggregation occurred in 14 instances. Of 34 videos of downward posture changes in sows, dispersion behavior was observed in 21 cases, and aggregation occurred in 6 instances. Comparison of the statistical results of aggregation and dispersion revealed a significant correlation between the piglet aggregation/dispersion behavior and the sow’s posture changes. Statistical correlations derived from 100 video samples are illustrated in Figure 8. The correlation values between piglets’ aggregation and dispersion and sows’ upward and downward posture changes are 0.6 and 0.82. The results demonstrate a significant correlation between piglets’ movement patterns (aggregation or dispersion) and sows’ posture changes (upwards or downwards).

## 4. Discussion

### 4.1. Performance Analysis of the Proposed Model for Piglet Tracking

**Figure 9:**
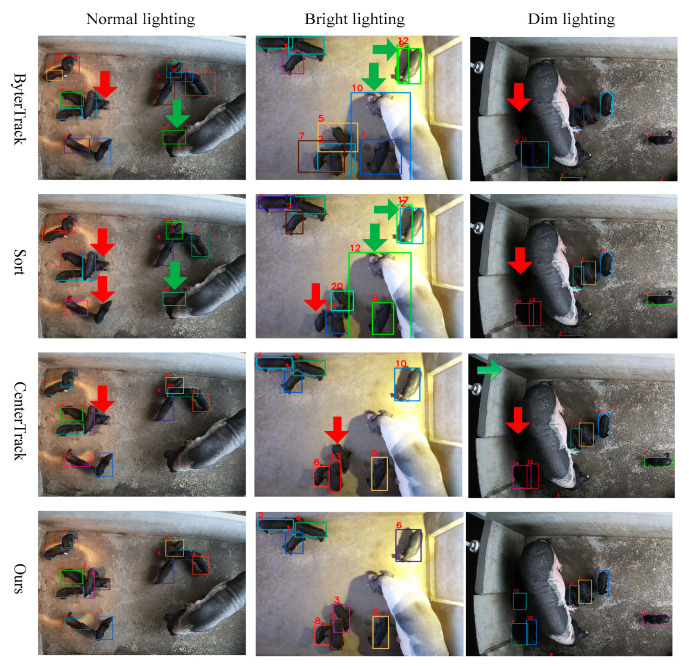
Visualization of tracking results of ByterTrack, Sort, CenterTrack, and the proposed method under normal-, bright-, and dim-lighting environments.

Some representative examples are selected to demonstrate the performance of our proposed piglet tracking model. We demonstrate its tracking results under various lighting conditions, including normal-lighting, bright-lighting, and low-lighting environments, compared with the ByteTrack, Sort, and CenterTrack methods. The visual results are shown in Figure 9. The red arrows point to false negatives, while the green arrows point to false positives. The number prefixed with “#” in the upper left corner of each image represents the frame ID. The colored rectangles represent the bounding boxes of pigs, with each color corresponding to a distinct individual. The number in the upper left corner of the bounding box indicates the target ID. Note that these symbols maintain consistent semantic representations across all subsequent figures. It can be observed that MSHMTracker has good robustness to lighting conditions. ByterTrack and Sort are sensitive to lighting variations and may falsely detect dark objects as targets, e.g., shadows or the dark ears of the sow. This can be attributed to two aspects of MSHMTracker: (1) using the heatmap as input enhances the judgment of object detection, reducing false positives, like the targets highlighted with green arrows; (2) the proposed STHM integrates detection scores and loss duration, enabling the aggregation of appearance features from the same instance across different frames in the temporal domain, which enhanced the ID association of targets with low scores caused by occlusion, crowding, and abnormal lighting.

**Figure 10:**
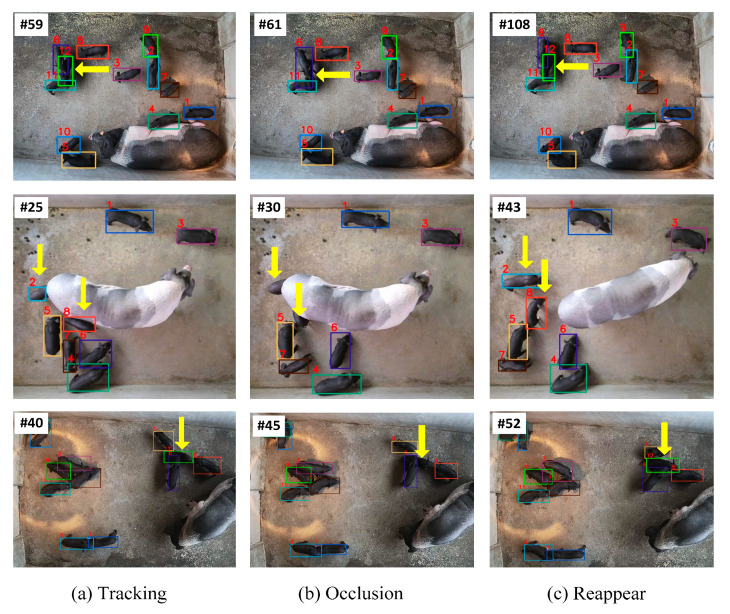
Visualization of occlusion examples.

We also selected several crowded and occluded samples to demonstrate the detection and tracking capabilities of our tracking model. The visualization results are shown in Figure 10. The yellow arrows highlight the occluded piglets. As shown in the first row of Figure 10, the piglet with ID12 was occluded by the piglet with ID6 in frame 61, while it was re-identified in frame 108. Similarly, in the second row, the piglets with ID2 and ID8 were occluded by the sow in frame 30, while they were re-identified in frame 43. In the third row, the piglet with ID12 was re-identified after it was occluded for seven frames. This can be attributed to the score–time-driven hierarchical matching mechanism STHM, which establishes spatio-temporal and appearance similarity associations to maintain identity continuity during occlusion transitions.

**Figure 11:**
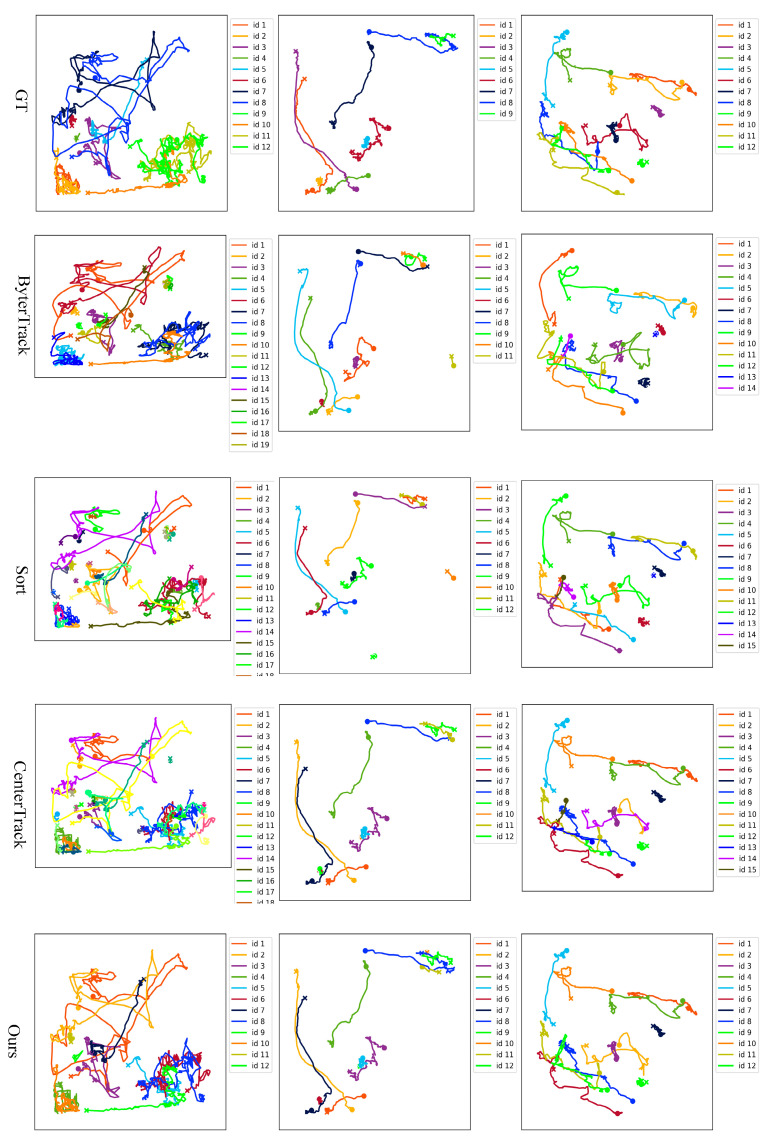
Visualization of piglets’ trajectories obtained with different methods. The colored lines present the trajectories of the pigs, with each color corresponding to a distinct individual.

Figure 11 presents the tracking trajectories of ByterTrack, Sort, CenterTrack, and our method to demonstrate the overall performance of video tracking. Different colored lines represent the motion trajectories of different targets. Each target is described in the legend. We present tracking trajectories from three video sequences, displayed in three columns, and the number of pigs in the three video sequences is 12, 9, and 12, respectively. It can be observed that the tracking trajectory with our method is the most similar to the real trajectory. Most methods in Figure 11 tend to assign new IDs when previously lost targets reappear during the tracking process, resulting in the total number of piglets being greater than the real number. Qualitative analysis of video sequence 2 reveals that our method still suffers from identity switches. The main reason is that significant posture variations and shape deformation occur after occlusion events, which simultaneously degrade both appearance-based re-identification and motion-based association performance.

### 4.2. Analysis and Exploration of Behavior Patterns Between Sow and Piglets

**Figure 12:**
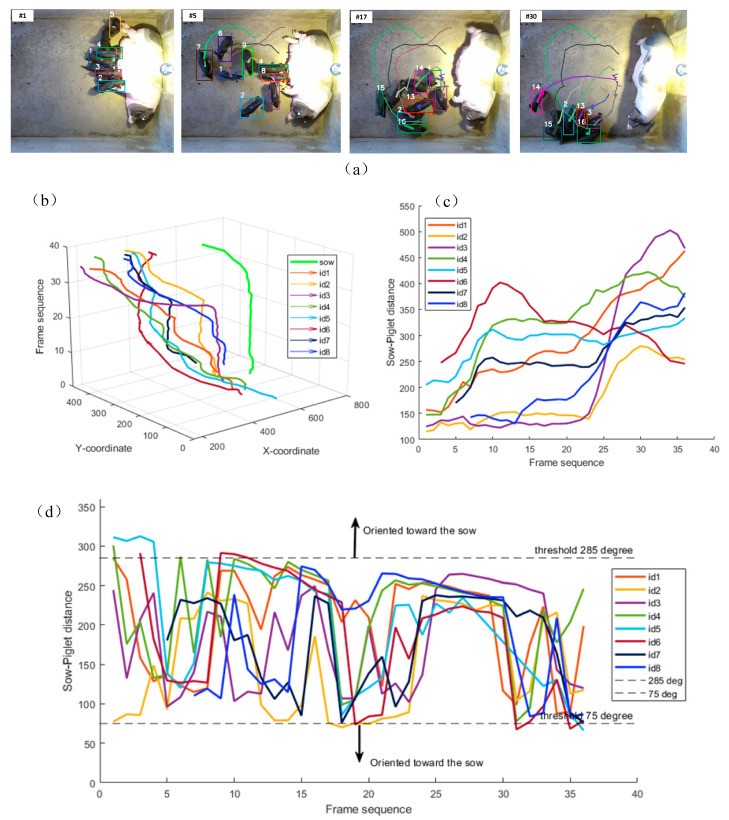
Visualization of piglet movement characteristics during the sow’s upward posture change. (**a**) The movement trajectories of the piglets. The colored lines present the trajectories of the pigs, with each color corresponding to a distinct individual. (**b**) The changes in the center points of the sow and piglets over time. (**c**) The changes in the distance between the sow and piglets over time. (**d**) The angular variation between the piglet movement direction and sow orientation over time. The angle reflects the piglet’s movement direction relative to the sow.

We selected a video clip of piglets gathering toward the sow and performed a visual analysis of their movement direction and relative distance to the sow. As can be seen from Figure 12, the distance between the sow and piglets gradually decreases over time, and the piglets’ movement direction is oriented toward the sow. In Figure 12, each target is described in the legend. These results demonstrate that the sow–piglet distance and the piglet’s movement direction are closely related to the behavior of the piglet group and are capable of distinguishing piglet aggregation/dispersion behaviors.

From the statistical results of piglets’ stress behavior responses to the sow’s posture changes, it is evident that the aggregation and dispersal behaviors of piglets are closely associated with changes in the sow’s posture. Under the sow’s upward posture changes, dispersion behavior predominated over aggregation behavior. This pattern likely occurs because most upward posture transitions involve the sow standing up, typically followed by locomotion. During this process, piglets tend to stay away from the sow to avoid being crushed. However, piglets tend to aggregate during the sow’s downward posture transitions. This phenomenon likely occurs because most downward postural transitions involve sows nursing or resting. During this process, piglets tend to gather around sows for breastfeeding, playing, or resting. Besides that, piglet aggregation/dispersion behaviors have no significant correlation with the rolling posture changes in sows, where the specific response of piglets usually depends on the intention of the mother sow. In addition, these behaviors are closely related to the maternal ability of sows, which can reflect their willingness to breastfeed, the quality of maternal care, and even their stress state. For instance, aggregation potentially facilitates offspring protection, reflecting a good maternal behavior, whereas a high dispersion frequency is often associated with inferior maternal ability.

### 4.3. Limitations and Potential Applications

#### 4.3.1. Limitations

**Figure 13:**
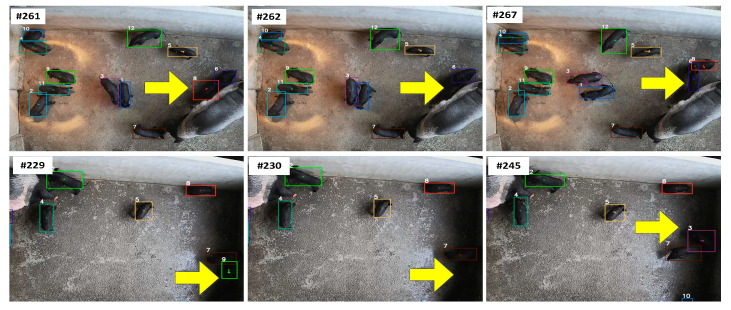
Examples of our tracking module’s failures.

While MSHMTracker has demonstrated robust performance in our experimental conditions, several limitations should be noted:

(1) The current validation was conducted exclusively on pig data collected from a single farm, limiting the generalizability of the findings. Although our multi-object tracking algorithm has shown good generalization across varying illumination conditions, occlusions, and high-density environments (see Section 4.1), its performance on other livestock species, pen structures, and atypical behavioral states has not been validated yet.
(2) Our tracking module still faces the challenges of ID loss and ID switching when piglets reappear but have undergone severe deformation or occlusion. Although we have implemented a temporary retention-and-iteration mechanism to address this issue, this approach is only effective when piglets regain their original shape or become non-occluded within a very short time frame. Moreover, the mechanism introduces non-negligible latency. Figure 13 presents several examples where our tracking module failed. At the top of Figure 13, occlusion led to the tracking failure of Target 2 in frame 262, followed by an ID switching event between Targets 2 and 3 in frame 267 due to severe shape deformation. Similarly, at the bottom of Figure 13, under low-light conditions, the tracking system lost Target 3 in frame 230 and incurred an identity mismatch in frame 245 due to a blurred appearance.

(3) The piglet group behavior recognition, the mean distance difference, and the most frequent movement direction of piglets were calculated to assess the individual behavior of piglets in the overall video. These metrics are sensitive to noise. For example, the unconscious wandering or body swaying of piglets may affect the overall mean distance difference and the most frequent movement direction due to the generated displacement and movement direction.

#### 4.3.2. Potential Applications

While this study has certain limitations, this work can provide information support for selective breeding and pig health management. In addition, the proposed multi-object tracking method and group behavior recognition approach provide novel insights for individual tracking and group behavior analysis in intensive farming environments. This work can be extended to several potential applications:

(1) The proposed tracking method can reduce ID losses and switches under crowding, occlusion, and deformation conditions, which can be used for other animals’ tracking.
(2) Our system can be expanded to maternal behavior detection, such as nursing refusal, hostile chasing, or aggression, by remodeling the trajectory information of individual movements.
(3) Individual-level tracking of individual stress behavior responses to sow posture changes could reduce piglet crushing. In future work, we will focus on individual-level monitoring to provide farmers with piglet-specific welfare indicators.

## 5. Conclusions

In this work, an enhanced JDT-based multi-object tracking method named MSHMTracker was proposed, which focuses on the spatio-temporal and appearance similarity association driven by motion status to maintain identity continuity during occlusion transitions. Extensive ablation experiments and comparison experiments were conducted on data from a real pig farm. All results demonstrate the superiority of MSHMTracker in terms of IDF1 (+2.1%) and MOTA (+2.3%). Further, the sow–piglet distance and the piglet’s movement direction relative to the sow were calculated and used to identify the piglet aggregation/dispersion behavior. The average accuracy of behavior recognition reached 87.49%. Finally, statistical analysis of the piglet aggregation/dispersion behavior under the sow’s posture changes demonstrates that the movement patterns (aggregation and dispersion) of piglets are closely related to the posture transition (upwards and downwards) of sows. This result lays the foundation for research on social relationships and behavioral patterns between lactating sows and piglets.

## Figures and Tables

**Table 1:** Description of dataset used for tracking and behavior analysis.

Dataset	Video	Pen
Training set	72	Pen 1, Pen 2, Pen 4, Pen 7, Pen 8, Pen 9, Pen 10, Pen 11
Validation set	8	Pen 1, Pen 2, Pen 4, Pen 7, Pen 8, Pen 9, Pen 10, Pen 11
Test set	30	Pen 3, Pen 5, Pen 6
**Sow behavior**	**Video**	**Description**
Upward posture change	52	Such as sitting-to-standing, lying-to-standing, lying-to-sitting
Downward posture change	34	Such as standing-to-sitting, standing-to-lying, sitting-to-lying
Rolling posture change	14	Such as ventral lying-to-lateral lying, lateral lying-to-ventral lying
**Piglet group behavior**	**Video**	**Description**
Aggregation	41	Over 50% of piglet population exhibits movement towards the sow
Dispersion	36	Over 50% of piglets exhibit movement away from the sow
Others	23	No obvious gathering or dispersing behavior

**Table 2:** Comparison of the proposed matching modules. FM, SM, and TM refer to the first, second, and third matching. A checkmark (√) indicates the method was adopted in the experiment, while a hyphen (-) means it was not employed. The arrow direction in the table shows a positive correlation between the metric value and performance quality.

Method			Evaluation Metrics		
FM	SM	TM	IDF1 ↑	MOTA ↑	IDs ↓
√	-	-	91.5%	91.6%	0.1%
√	√	-	92.5%	94.1%	0.2%
√	√	√	92.9%	93.8%	0.1%

**Table 3:** The tracking rate of each piglet in all of our test data.

Video ID	Piglet ID											
	1	2	3	4	5	6	7	8	9	10	11	12
1	1	1	1	1	1	1	1	1	1	1	0.991	0.981
2	0.97	1	0.99	1	1	1	1	1	-	-	-	-
3	0.92	1	1	0.96	1	1	1	1	1	0.82	1	1
4	1	1	1	1	1	1	0.9812	0.975	-	-	-	-
5	1	1	0.99	1	0.99	1	1	1	-	-	-	-
6	0.94	1	0.94	1	1	0.961	1	0.99	1	-	-	-
7	1	0.94	0.99	1	1	0.99	0.99	0.99	0.99	1	1	0.99
8	1	1	1	1	1	1	1	1	0.99	0.97	1	0.96
9	1	0.99	0.98	1	0.76	1	1	1	1	0.911	0.98	0.981
10	1	1	1	1	1	0.99	1	1	-	-	-	-
11	1	1	1	0.99	1	1	1	1	1	1	1	1
12	1	1	1	1	1	0.96	1	1	-	-	-	-
13	1	1	1	1	1	1	1	1	1	1	0.95	1
14	0.99	1	1	1	1	1	1	0.99	-	-	-	-
15	1	0.941	1	0.98	0.97	1	0.91	-	-	-	-	-
16	0.99	1	1	1	1	1	0.99	1	-	-	-	-
17	1	1	1	1	1	1	1	1	1	1	1	1
18	1	0.99	1	1	1	0.95	1	0.85	1	1	0.99	0.89
19	1	0.99	1	1	1	1	1	1	0.94	-	-	-
20	1	1	1	0.99	1	0.94	1	0.98	-	-	-	-
21	1	1	1	1	1	1	1	0.99	1	0.951	0.58	-
22	1	1	1	1	0.97	1	1	1	1	1	1	1
23	0.86	1	1	1	0.99	1	1	1	1	1	0.99	1
24	1	1	1	1	1	1	0.99	0.954	-	-	-	-
25	0.99	1	1	1	1	1	1	0.902	1	1	1	0.77
26	1	1	1	1	1	1	1	1	-	-	-	-
27	0.69	0.99	1	1	1	0.99	0.91	1	1	1	0.994	0.761
28	1	0.96	1	1	1	0.99	0.94	1	0.04	0.861	0.781	1
29	1	1	1	1	1	1	1	1	1	1	1	0.98
30	0.91	1	1	1	1	1	0.99	0.96	1	0.99	0	1

**Table 4:** Comparison among different tracking methods on our test dataset. The arrow direction in the table shows a positive correlation between the metric value and performance quality.

Methods	IDF1 ↑	MOTA ↑	IDs ↓	FP ↓	FN ↓
Motdt	55.1%	84.7%	4.7%	5.7%	4.9%
FairMot	61.7%	86.6%	2.3%	4.6%	6.6%
DeepSort	74.2%	80.1%	1.1%	10.4%	9.4%
ByterTrack	86.6%	79.1%	0.1%	11.0%	9.8%
Sort	87.6%	90.4%	0.4%	5.7%	3.6%
Centertrack	90.8%	91.5%	0.1%	6.0%	2.4%
MSHMTracker (Ours)	92.9%	93.8%	0.1%	2.4%	3.4%

**Table 5:** The results of behavior recognition on piglet group.

Recognition	Ground Truth		
	Aggregation	Dispersion	Others
Aggregation	37	1	0
Dispersion	0	26	0
Others	4	9	23
Accuracy/%	90.24	72.22	100.00
Total accuracy/%	87.49%		

**Table 6:** The statistical results for piglet aggregation/dispersion behavior under different sow posture transitions.

Classification of Sow Posture Changes	Aggregation Videos (95% CI)	Dispersion Videos (95% CI)	Others Videos (95% CI)
Upward posture changes	14 [8, 20]	27 [20, 34]	11 [5, 17]
Downward posture changes	21 [16, 27]	6 [2, 10]	7 [2, 12]
Rolling posture changes	6 [3, 10]	3 [1, 6]	5 [2, 9]

## Data Availability

The datasets used in this study are available from the corresponding author upon reasonable request.

## Abbreviations

The following abbreviations are used in this manuscript:

STHM	Score- and time-driven hierarchical matching mechanism
JDT	Joint detection and tracking
MSHMTracker	Motion-status-driven hierarchical multi-object tracking method
MOT	Multi-object tracking
